# Validation of optical voltage reporting by the genetically encoded voltage indicator VSFP-Butterfly from cortical layer 2/3 pyramidal neurons in mouse brain slices

**DOI:** 10.14814/phy2.12468

**Published:** 2015-07-29

**Authors:** Ruth M Empson, Chelsea Goulton, David Scholtz, Yasir Gallero-Salas, Hongkui Zeng, Thomas Knöpfel

**Affiliations:** 1 Department of Physiology, Brain Health Research Centre, Otago School of Medical Sciences, University of OtagoDunedin, New Zealand; 2 The Division of Brain Sciences, Department of Medicine, Imperial College LondonLondon, United Kingdom; 3 Allen Institute for Brain ScienceSeattle, Washington

**Keywords:** Cortex, genetically encoded voltage indicator, L2/3, optogenetics, voltage imaging

## Abstract

Understanding how behavior emerges from brain electrical activity is one of the ultimate goals of neuroscience. To achieve this goal we require methods for large-scale recording of the electrical activity of specific neuronal circuits. A very promising approach is to use optical reporting of membrane voltage transients, particularly if the voltage reporter is genetically targeted to specific neuronal populations. Targeting in this way allows population signals to be recorded and interpreted without blindness to neuronal diversity. Here, we evaluated the voltage-sensitive fluorescent protein, VSFP Butterfly 2.1, a genetically encoded voltage indicator (GEVI), for monitoring electrical activity of layer 2/3 cortical pyramidal neurons in mouse brain slices. Standard widefield fluorescence and two-photon imaging revealed robust, high signal-to-noise ratio read-outs of membrane voltage transients that are predominantly synaptic in nature and can be resolved as discrete areas of synaptically connected layer 2/3 neurons. We find that targeted expression of this GEVI in the cortex provides a flexible and promising tool for the analysis of L2/3 cortical network function.

## Introduction

The last few years has seen tremendous advances in our ability to optically image electrical activity in neurons (Scanziani and Hausser 2009) using genetically encoded fluorescence-based voltage and calcium indicators (GEVIs and GECIs) (Akemann et al. 2010; Jin et al. 2012; Knopfel 2012; Chen et al. 2013; St-Pierre et al. 2014). The development of these optogenetic monitoring tools is driven by our desire to understand how connectivity among specific populations of neurons supports brain network activity and the generation of behavior. Achieving this goal is a major challenge for neuroscience and requires GEVIs and GECIs that can robustly monitor neuronal activity across the time scales of action potentials (a few milliseconds) and slower synaptic potentials (tens to hundreds of milliseconds) and with cellular level spatial resolution. Equally important is the development of rodent models that facilitate neuron class-specific expression of these optical reporters.

To meet these requirements we have recently used an intersectional genetic targeting strategy based on the new expression locus TIGRE (TITL) to specifically express the GEVI called Voltage Sensitive Fluorescence Protein (VSFP) Butterfly 2.1 (Akemann et al. 2012) at robustly high levels in layer (L) 2/3 cortical pyramidal neurons of the mouse cortex (Madisen et al. 2015). Previously, expression of Butterfly in cortical neurons using in utero electroporation successfully reported strong voltage signals in mouse sensory cortex during anesthesia and wakefulness (Akemann et al. 2012; Scott et al. 2014; Carandini et al. 2015; Madisen et al. 2015). When the membrane depolarizes, Butterfly exhibits an increase in voltage-dependent Förster resonance energy transfer (FRET) between a pair of yellowish-green and red fluorophores: mCitrine (donor) and mKate2 (acceptor). By imaging the emission from both fluorophores and taking the ratio of their fluorescence (mKate2/mCitrine), Butterfly reports the membrane voltage of activated neurons (Akemann et al. 2012).

This study took advantage of the in vitro brain slice preparation to validate the voltage reporting performance of Butterfly and its ability to monitor electrical activity in L2/3 from Rasgrf2-2A-dCre;Camk2a-tTA;Ai78 (TITL-VSFPB) Butterfly expressing mice (Madisen et al. 2015). Both standard widefield fluorescence imaging and two-photon (2P) imaging provided robust membrane voltage recordings from L2/3 neurons. Pharmacological approaches confirmed that Butterfly successfully reported action potential and subthreshold synaptic events in L2/3. Subthreshold events were also detected by Butterfly as discrete areas of synaptically connected L2/3 neurons. While the development of mouse lines that express GEVIs in specific classes of neurons is motivated by our desire to perform chronic in vivo voltage imaging in behaving animals, our present study validates this approach and also highlights the promising features of these tools for the analysis of functional connectivity within L2/3 cortical networks in vitro.

## Methods

### Mice

Male and female triple transgenic Rasgrf2-2A-dCre;Camk2a-tTA;Ai78 mice (obtained from the Allen Institute for Brain Science) expressing CRE, tTA, and VSFP Butterfly were selected based upon PCR-based genotyping from genomic DNA using the following primers.

CRE 5′-ACCCTGTTACGTATAGCCG-3′ Forward.

5′-GAGTCATCCTTAGCGCCGTA-3′ Reverse.

tTA 5′- CAACCCGTAAACTCGCCCAGAAG -3′ Forward.

5′-GGCCGAATAAGAAGGCTGGCTCT -3′ Reverse.

Butterfly 5′-TCAAGGAGGCCGACAAAGAGACC -3′ Forward.

5′-ACAACCAACTGCCCCAAACCATC -3′ Reverse.

Recombinase activity of dCre (and Butterfly expression) is induced with the antibiotic trimethoprim (TMP) in 10% DMSO (Sigma, Auckland, New Zealand) (Sando et al. 2013) delivered by intraperitoneal injection (250 mg/kg) over 3 consecutive days or in the food as a raspberry jelly (10 mg TMP in 10% DMSO available each day) for a total of 7 days with prior familiarization to vehicle-only jelly (Zhang 2011). 14 days to 4 months after induction we prepared 300-*μ*m-thick slices from the motor cortex and neocortex of mice (Tantirigama et al. 2014). Slices were transferred to the stage of a Nikon Eclipse FN-1 microscope and continually perfused with artificial cerebrospinal fluid (ACSF) containing (in mM) 126 NaCl, 3 KCl, 1 NaH_2_PO4, 2 MgSO_4_, 2 CaCl_2_, 25 NaHCO_3_, 15 glucose, equilibrated with 95% O_2_ and 5% CO_2_ at room temperature (22–24°C). All animal manipulations were previously approved by the University of Otago Animal Ethics Committee and the Home Office, UK who both work to international ethical guidelines for the use of animals in research.

### Electrophysiological recordings and imaging

Local field potential recordings (×1000 amplification, Neurolog NL109, Digitimer, UK/Axopatch 200B/Multiclamp 700B, Molecular Devices, Sunnyvale, CA) collected using Signal (CED, UK) or pClamp10 (Molecular Devices) determined the health and viability of the slices prior to imaging. We did not observe prolonged or epileptiform activity in any of our slices.

Single, 1× and repeat, 5× (50 Hz) electrical stimulation (constant positive polarity voltage, 100–200 *μ*sec duration, Digitimer DS2A, Iso-Flex AMPI, Israel) used a monopolar-stimulating electrode (1–1.5 MΩ, resistance) placed remotely (4–500 *μ*m away but in the cortical column, or closer in L2/3 for 2-photon [2P] imaging) from the recording electrode and imaging area. Standard widefield fluorescence imaging and electrical recordings were synchronized with a Master8 (AMPI) and images collected at 100 Hz using a high speed camera (Hamamatsu, Japan) and Simple PCI software using either a 16× or 60× immersion objective (NA 0.8 and 1.0, respectively) and with Semrock (Rochester, NY) filters designed to capture mCitrine and mKate2 emission following excitation of mCitrine using shutter controlled light from a 150W Moritex MHAB lamp. Optical filters and dichroic mirrors used: FF01-483/32-25 for mCitrine excitation; FF510-Di02-25x36 510 LP for excitation/emission light separation; FF580-FDi01-25x36 dichroic mirror for mCitrine/mKate2 emission light separation; and 580LP BLP01-594R-25 mKate2 emission filter, all Semrock.

For 2P imaging we scanned (Ultima IV, Bruker, Coventry, UK) at 25 Hz using a Ti-sapphire Laser (Chameleon Coherent, Ely, UK) set at 920 nm with a 16× Nikon objective (NA 0.8). Emission light was separated from excitation light by a dichroic mirror (Multiphoton-LP-Beamsplitter 720 DCXXR, Chroma, Bellows Falls, VT) and filtered with an infrared blocking filter (ET700sp-2p8, Chroma). mCitrine and mKate2 emission was separated as above and filtered with FF01-542/50-25 and BLP01-594R-25 (Semrock), respectively.

All images were corrected for bleaching using a linear correction based upon nonstimulated imaging responses and we averaged 3–5 sweeps for all recordings. Regions of interest within L2/3 were either 250–300 *μ*m × 250–300 *μ*m at low magnification (16× objective) or 30 *μ*m × 30 *μ*m at higher magnification (60× objective). Signals from 30 *μ*m squares were compared by normalizing their peak to the SD of their baseline noise; signals greater than 2SD of the baseline noise were defined as activated areas. 2P image sequences are averaged (25 trials), spatially filtered (Gauss spatial filter size 3, strength 5), changes in mCitrine and mKate2 fluorescence (Δ*F*/*F*_0_) and their ratio (Δ*R*/*R*_0_) calculated using Matlab7 and ImagePro 6.2.

Pharmacological blockade of AMPA and NMDA receptor-mediated excitatory synaptic responses used ACSF supplemented with (in *μ*M) 20 CNQX, 50 D-APV, and 1 tetrodotoxin, TTX (Invitrogen, Penrose, New Zealand) to block voltage gated Na^+^ channels applied to the slices for 10–15 min.

Statistical analysis, one-way ANOVA and curve fitting were calculated in Prism using a significance of *P* < 0.05.

### Post hoc immunohistochemistry and butterfly detection

Butterfly expressing free-floating slices from the imaging experiments were fixed in 4% paraformaldehyde in phosphate-buffered saline (PBS) overnight before permeabilization (0.2% Triton X-100) and blocking with 5% goat serum (Sigma) in PBS. Overnight exposure to antibodies to vGlut2 (Synaptic Systems, Goettingen, Germany, Cat. No. 135403, rabbit) and CUX1 (Santa Cruz, Dallas, TX, Cat. No. M-222, rabbit) was followed by secondary detection (4 h) using an anti-rabbit Alexa647 coupled secondary antibody (Invitrogen) all in PBS. No specific staining was observed in the absence of primary antibody. Slices were visualized with 10×, NA 0.3 and 40×, NA 0.75 objectives with a Nikon A1R confocal microscope using 488 nm and 635 nm laser lines (Coherent Scientific, Hilton, SA, Australia) for excitation of Butterfly and Alexa 647, respectively. Fluorescence was detected through band pass filters 525 ± 50 and 630 ± 50 nm).

## Results

### Butterfly is reliably expressed in layer 2/3 of the cortex

**Figure 1:**
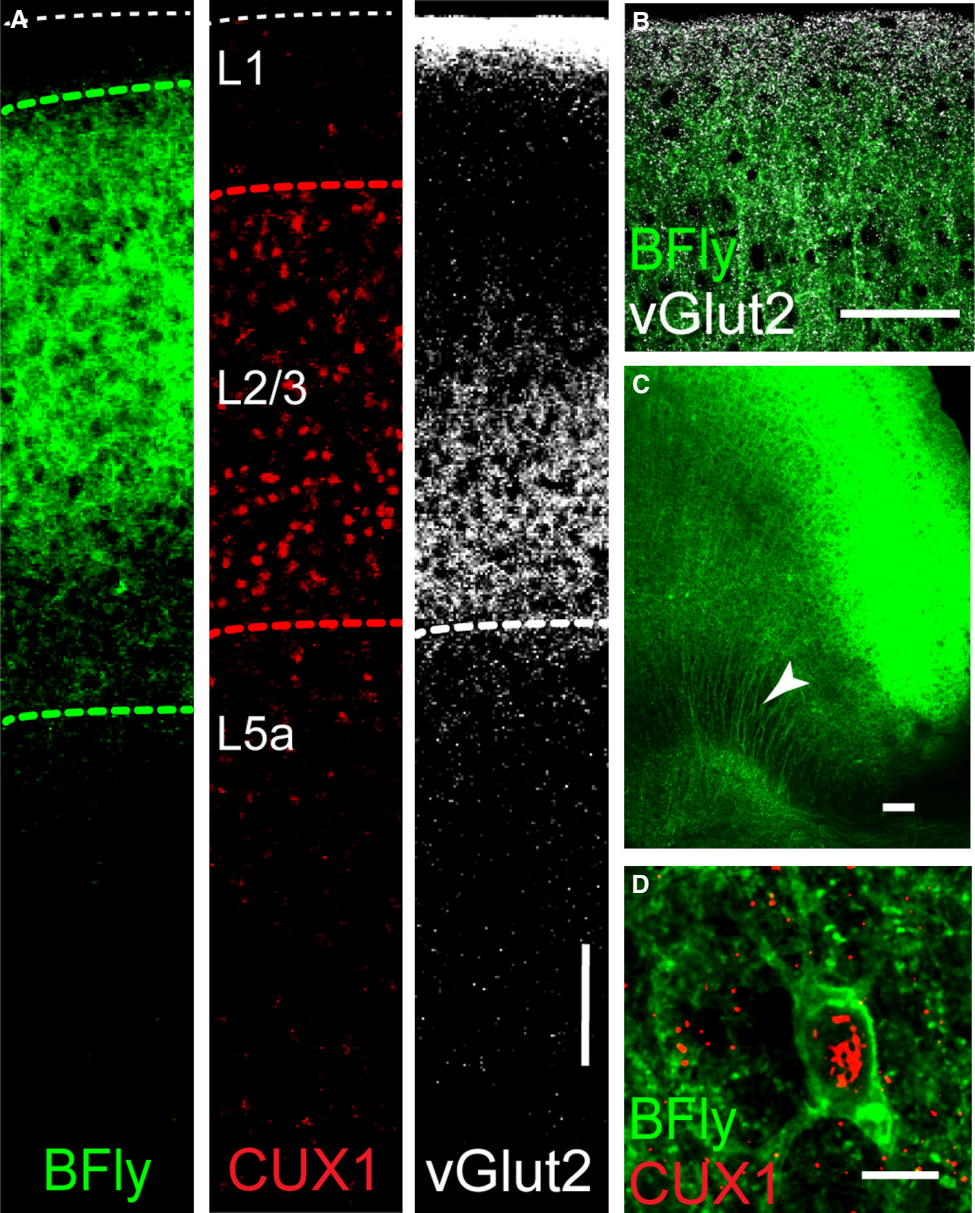
Butterfly VSFP2.1 is expressed in L2/3 pyramidal neurons of motor cortex from the Rasgrf2-2A-dCre;Camk2a-tTA;Ai78 (TITL-VSFPB) Butterfly mouse. (A) Layer specific Butterfly expression in the motor cortex evident within the boundaries occupied by L2/3 pyramidal neuron somas and dendrites, green dashed lines, L2/3 is defined by the presence of CUX1-expressing pyramidal neurons (red dashed lines) and its lower boundary with L5a defined by vGLUT2 expression (white dashed line, scale bar 100 *μ*m). Butterfly expression in L2/3 pyramidal neuron apical dendrites among vGLUT2-positive terminals in L1, (B) L2/3 pyramidal neuron axon projections (arrowhead), (C) (scale bar 100 *μ*m), and the somatic plasma membrane, (D) (scale bar 10 *μ*m).

Low magnification inspection of slices from Rasgrf2-2A-dCre;Camk2a-tTA;Ai78 mice (these triple transgenic mice are subsequently referred to as Butterfly mice) revealed very bright fluorescence throughout all areas of the cerebral cortex and specific labeling of the superficial layers. Butterfly expression coincided with the location of CUX1 expression, a homeodomain family of DNA-binding proteins that identifies L2/3 pyramidal neuron nuclei (Nieto et al. 2004). Note that Butterfly expression extends beyond the CUX1-positive L2/3 cell bodies (green versus red dashed lines in Fig.1A) consistent with its expression in the apical (Fig.1B) and basal dendrites of the pyramidal neurons. Butterfly expression also remained within the boundaries of vesicular glutamate transporter type 2 (vGlut2)-expressing terminals (Hirai et al. 2012) in layer 1 and at the boundary of L2/3 and L5a of motor cortex (Fig.1A). We did not observe significant Butterfly fluorescence in other layers although we could visualize L2/3 pyramidal neuron axons passing through layer 5 toward the white matter (Fig.1C, arrowhead). Higher magnification confirmed the specific membrane location of Butterfly in the somatodendritic membranes of CUX1-positive layer 2/3 neurons (Fig.1D) with very little evidence of internal cytoplasmic Butterfly fluorescence (Perron et al. 2009).

### Butterfly robustly reports population responses from L2/3 following electrical stimulation

**Figure 2:**
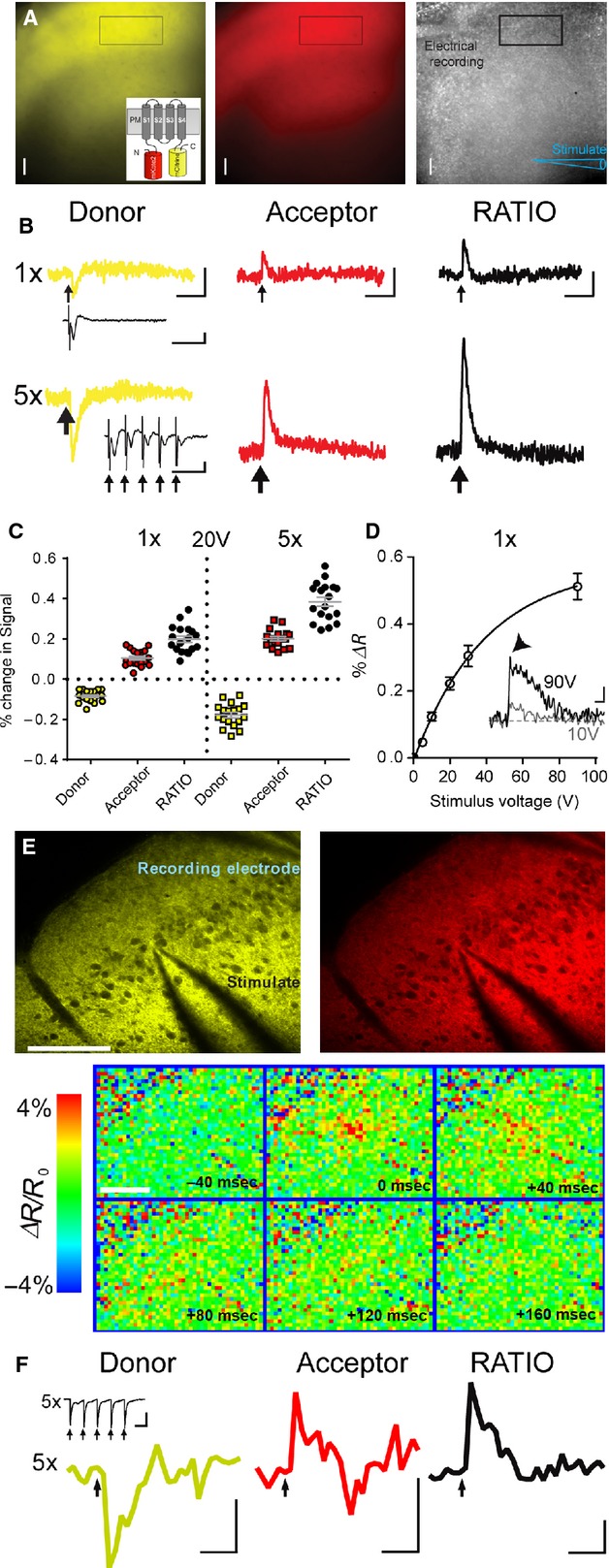
Butterfly VSFP 2.1 robustly monitors voltage transients from populations of L2/3 pyramidal neurons in response to electrical stimulation. (A) Widefield images of Butterfly expression, mCitrine, yellow and mKate2, red, in a living slice (spatial scale bar 100 *μ*m) with position of electrical stimulation and recording electrodes; inset shows a schematic of Butterfly FRET pairs. (B) Widefield imaging responses from Butterfly mCitrine (donor) and mKate2 (acceptor) (average of 10 sweeps, scale bars 1 sec; 0.1% Δ*F*/*F*_0_ and Δ*R*/*R*_0_ apply to both 1× and 5× responses), from a region of interest placed in L2/3 (rectangle in A), in response to 1× and 5× electrical stimulation (20 V, downward arrows) with accompanying LFPs, scale bars 0.1 mV; 50 ms. Note the decrease in donor fluorescence and increase in acceptor fluorescence as expected from modulation of FRET efficacy. (C) Summary data from seven animals illustrating the robust and reproducible nature of the optical readout, all individual values are shown with mean ± SEM in gray. (D) Butterfly signal as a function of stimulation intensity, values are mean ± SEM (data is from 5 to 8 slices at each stimulation intensity). Inset is an example recording, note the fast (arrowhead) and slow components of the Butterfly signal at high stimulation intensities, scale bars 100 ms; 0.2% Δ*R*/*R*_0_. (E) Imaging of electrical stimulation-induced voltage transients using two-photon microscopy mCitrine (left, yellow) and mKate2 fluorescence (right, red); stimulating (center) and recording electrode (top right corner), scale bar 100 *μ*m. Lower panel shows color-coded sequence of voltage maps 40 ms before to 160 ms after stimulation (*t* = 0, 5 pulses, 0.2 ms, 100 Hz). Depolarization initiates close to the stimulating electrode tip and then spreads to nearby areas. (F) Donor (mCitrine), Acceptor (mKate2), and Ratio (mKate2/mCitrine) fluorescence transients from the region of interest above (scale bars 1% Δ*F*/*F*_0_ and Δ*R*/*R*_0_; 250 ms). Black arrows indicate time of electrical stimulation. Traces represent average over 25 trials. Inset shows associated LFP, scale bars 1 mV; 10 ms.

Regions of interest representing large numbers of Butterfly positive L2/3 neurons in motor cortex slices (detected with a 16× objective) responded reliably and robustly to remote electrical stimulation (Fig.2). Decreases in the brightness of the donor, mCitrine, accompanied by increases in the brightness of the acceptor, mKate2, indicated reliable modulation of FRET between the two fluorophores in response to excitation (depolarization) of the L2/3 neurons. In all cases a single stimulation (1×, 10–20 V) was sufficient to reveal a clearly detectable fluorescence response above baseline in single sweeps (both mCitrine and mKate) with little bleaching (mCitrine 0.086 ± 0.03% per sec; mKate2 0.079 ± 0. 03% per sec, 16× objective, *n* = 9 slices from 4 animals) that could be recorded for many hours with no signs of toxicity or degradation of fluorescence or electrophysiological signals even after prolonged imaging. Local field potentials (LFPs) provided an independent verification of electrical synaptic activity within the slice preparation. Repetitive stimulation (5× stimuli at 50 Hz) reliably doubled the size of the fluorescence transients due to the integrative properties of VSFP Butterfly 2.1 (Akemann et al. 2012) but note that our image sampling frequency of 100 Hz precludes identification of responses to individual stimuli delivered at 50 Hz. Note also the depression of the LFP amplitude during 5× stimulation (inset Fig.2B).

The amplitude of Butterfly responses following a single stimulation, 1×, increased as a function of stimulation voltage between 10 and 40 V (Fig.2D). Stimulation intensities above 20 V usually evoked a fast antidromic spike prior to the slower synaptic component of the LFP and both were accompanied by a fast initial, and longer slower, component of the Butterfly signal (inset to Fig2D).

We recorded LFPs (not shown) from the cortex of nontriple transgenic litter mate mice (*n* = 2) that were similar to those seen in the triple transgenic mice. Using the same excitation and emission wavelengths employed to detect Butterfly signals we tested for stimulus-evoked intrinsic fluorescence signals from nontriple transgenic mice. We observed very small, slowly rising and long-lasting fluorescence transients that only occurred in response to a high-frequency (100 Hz) burst of 10 stimulations (data not shown, *n* = 2; see [Coutinho et al. 2004]).

2P imaging of Butterfly to detect voltage transients benefits from deeper tissue penetration, higher spatial resolution and larger Δ*R*/*R*_0_ values (Akemann et al. 2013). Here, electrical stimulation evoked optical responses similar to those obtained using widefield imaging above but provided higher spatial resolution throughout the neuropil (Fig.2E) and larger Δ*F*/*F*_0_ values (Fig.2F).

### Butterfly reports synaptic and action potential-mediated L2/3 activity

**Figure 3:**
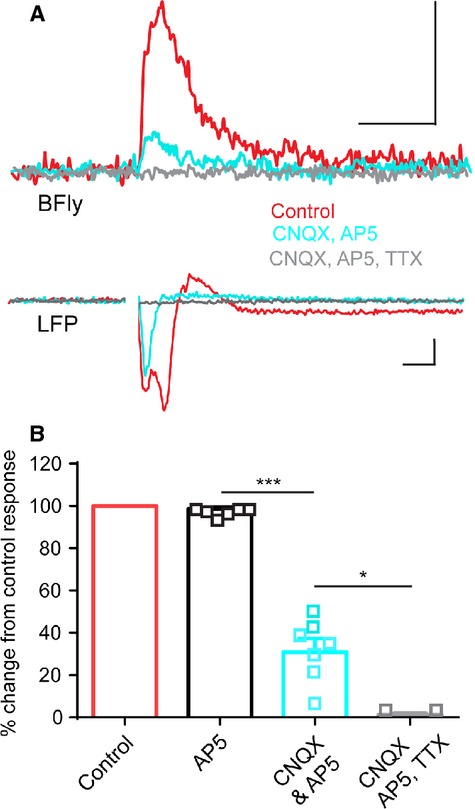
Electrical stimulation-induced Butterfly VSFP2.1 signals from the layer 2/3 population are predominantly synaptic but also contain a fast action potential-mediated component.(A) High stimulation voltage (90 V) -induced Butterfly response integrated over a large region of L2/3 (16× objective, 300 *μ*m × 300 *μ*m, average of five sweeps, widefield imaging) showing an initial fast component followed by a slower, longer lasting component (scale bars 0.5% Δ*R*/*R*_0_, 500 ms). The LFP exhibits a fast antidromic action potential and slower synaptic response (scale bars 0.1 mV, 5 ms), stimulus artifacts are removed. (B) pharmacological treatment with the excitatory glutamatergic synaptic blockers CNQX and APV reduces the Butterfly signal, leaving a TTX sensitive component remaining. Error bars are mean ± SEM and *** and * represent *P* < 0.001 and *P* < 0.05 (one-way ANOVA).

Butterfly is expressed in both the somatodendritic and axonal plasma membranes (Fig.1) raising the question of whether the observed voltage signals represent synaptic or axonal voltage transients. To address this question we combined widefield imaging with a pharmacological approach. Using stimulation intensities that evoked a fast antidromic spike and slower LFP (Fig.3) application of the excitatory amino acid receptor antagonists APV and CNQX together significantly reduced the amplitude and duration of the L2/3 Butterfly reported voltage transients (one way ANOVA, *P* < 0.001, Fig.3B) and the LFP, leaving approximately 30% of the Butterfly voltage transient remaining and the fast antidromic spike in the LFP largely intact. The APV- and CNQX-resistant component of the Butterfly reported voltage transient exhibited a fast rise time (10 ms or less) and was abolished by blockade of voltage gated Na^+^ channels with TTX. APV alone did not influence the Butterfly response or LFP. These findings validate Butterfly responses as a reliable monitor of both action potential and subthreshold synaptic responses from populations of L2/3 neurons, and that the larger portion of the optical voltage signal represents glutamatergic AMPA receptor-mediated synaptic events.

### Butterfly reports functional synaptic area connectivity within the L2/3 network

**Figure 4:**
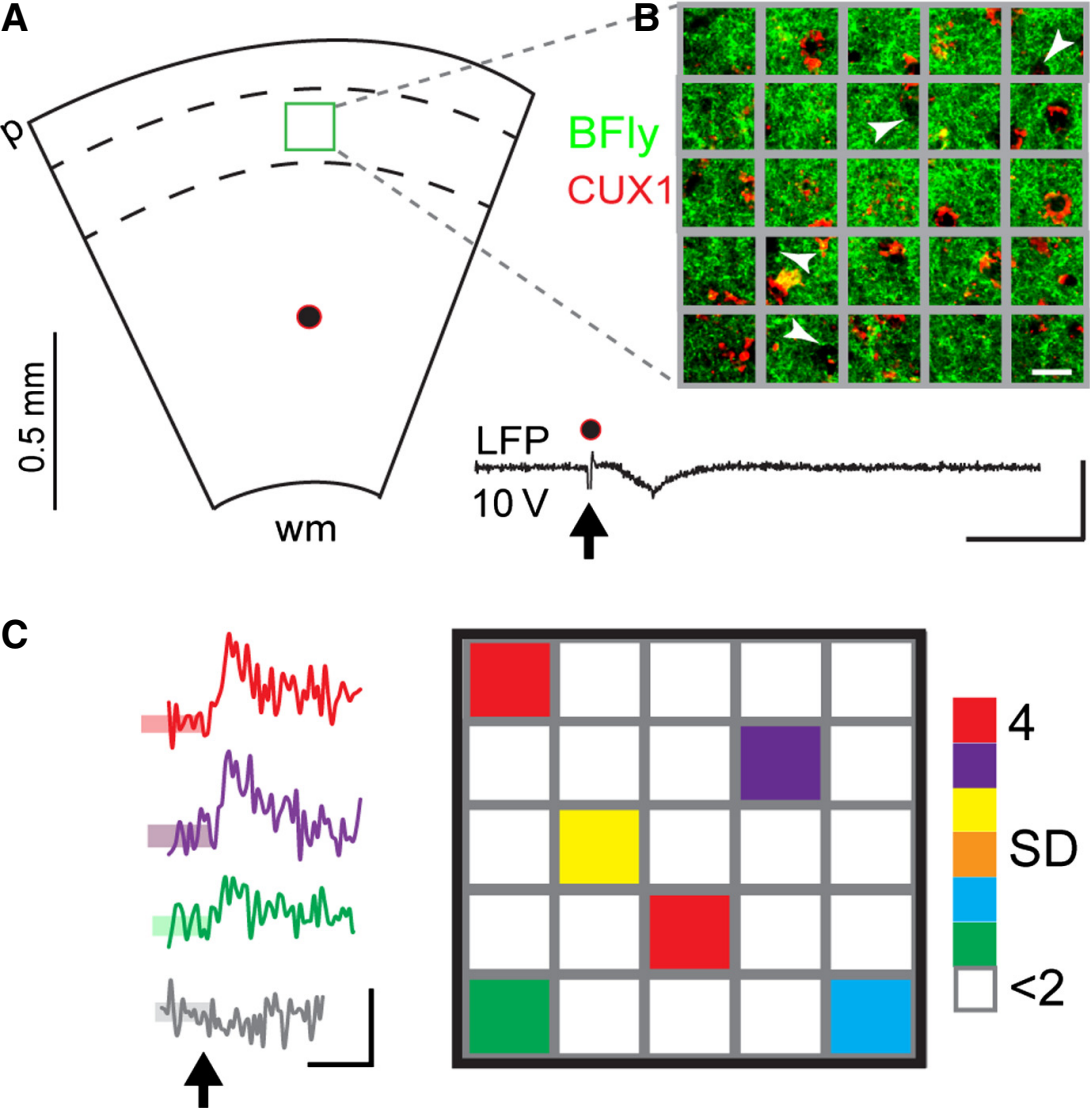
Butterfly VSFP2.1 successfully monitors discrete areas of sub-threshold excitatory synaptic connectivity among L2/3 pyramidal neurons. (A) Schematic of the remote-within-column minimal stimulation and 150 × 150 *μ*m field of view (green box) of the 60× objective, wm, white matter; p, pia, divided further into twenty-five 30 *μ*m × 30 *μ*m grid square areas (scale bar 15 *μ*m). (B) shows the location of Butterfly expressing CUX1-positive L2/3 neurons within the grid squares. Note also the CUX1-negative, Butterfly negative areas, white arrowheads, that are likely to represent inhibitory interneurons within L2/3. Minimal stimulation (10 V, arrow) evokes a small LFP, inset (scale bars 20 ms; 0.1 mV) and Butterfly responses (mKate only) from discrete areas, from an example in (C) where grid squares are color-coded for *z*-score Butterfly response amplitudes exceeding 2SD (SD of the baseline noise shown by the transparent colored boxes overlaid on the trace baselines on left). Color-coded traces show the time courses of responses to minimal stimulation, arrow, observed in the colour-coded areas in the right-hand panel (average of three sweeps, scale bars 0.1% Δ*F*/*F*_0_; 500 ms); gray trace is typical of responses observed in white colored areas <2SD of baseline noise.

Using a widefield imaging approach at higher spatial resolution (60× objective, 150 *μ*m × 150 *μ*m) we further examined the ability of Butterfly to monitor the spatial distribution of subthreshold synaptic responses among the interconnected L2/3 network in the slice. To do this we used a minimal intensity stimulation (10 V) remote from the imaging area, but within the cortical column, to evoke a small, subthreshold LFP in L2/3 (Fig.4) and measured Butterfly fluorescence responses from within discrete 30 *μ*m × 30 *μ*m areas of the interconnected L2/3 network. In response to the minimal stimulation Butterfly reported small amplitude increases in fluorescence (>2SD of baseline noise) from between 1 and 8 (out of 25) of these 30 *μ*m × 30 *μ*m areas in each slice, representing approximately 22 ± 0.04% of the monitored area (*n* = 7 slices from 5 animals). CNQX reduced these signals to less than 2SD of their baseline noise (*n* = 4 slices from 2 animals, 23 regions of interest) suggesting that these small amplitude Butterfly responses in the slice were exclusively synaptic in nature.

## Discussion

Using cortical brain slices we show that the genetically encoded voltage indicator VSFP Butterfly 2.1 targeted specifically to L2/3 pyramidal neurons in a transgenic mouse robustly monitors action potential and synaptically mediated population responses with good spatial and temporal resolution.

Butterfly was faithfully expressed in CUX1-positive L2/3 cortical pyramidal neurons within vGlut2-positive boundaries (Fig.1). This expression pattern is consistent with the intersectional genetic targeting strategy using CamKinaseIIa expressed in cortical pyramidal neurons (Erondu and Kennedy 1985) and *rasgrf2* expression in L2/3 pyramidal neurons (Allen Institute Mouse Brain Atlas http://mouse.brain-map.org/experiment/show/69817988). At higher magnification we detected Butterfly in the somatic membrane (Fig.1) of CUX1-positive L2/3 soma (Nieto et al. 2004), in their apical dendrites that extend into and toward L1, and also in their axons projecting out of L2/3 into L5.

Using standard widefield fluorescence and 2P imaging, Butterfly robustly reported the membrane voltage transients of the population of L2/3 neurons with excellent signal-to-noise ratio. The strong TRE promoter used in the TIGRE-based genetic targeting strategy (Madisen et al. 2015) drives high expression levels of Butterfly and improved the signal-to-noise ratio obtained with 2P imaging when compared with previous results using in utero electroporated mice (Akemann et al. 2013).

Butterfly voltage transients increased in amplitude with increasing stimulation intensity (Fig.2) and were largely synaptic in nature (Fig.3). Our results accord with previous observations using small molecular weight voltage-sensitive dye imaging in the superficial layers of the neocortex (Holmgren et al. 2003). In addition Butterfly revealed small, but fast rising TTX-sensitive signals when synaptic transmission was blocked. These signals most likely arise from a combination of antidromic activation of L2/3 somas following stimulation of their axons in L5, and activation of Butterfly expressing horizontal and reciprocally connecting axons among L2/3 cortical pyramidal neurons. These horizontal connections were previously identified with classical electrophysiology in vitro (Aroniadou and Keller 1993) and more recently with the GECI GCaMP3 in vivo (Vanni and Murphy 2014).

Widefield imaging with Butterfly could also detect sparse and spatially discrete subthreshold excitatory synaptic responses among the L2/3 network in the slice (Fig.4). Synaptic interactions between L2/3 neurons, though sparse, can be strong, (Petersen and Crochet 2013) and are within the detection range of Butterfly (Akemann et al. 2012). The spatially discrete signals observed here may represent electrically triggered synaptic network activity among functionally clustered L2/3 neurons perhaps via activation of the low probability synaptic connections between L5 and L2/3 (Lefort et al. 2009).

In summary, these in vitro findings validate reliable and specific functional expression of VSFP Butterfly 2.1 exclusively in the soma, dendrites and axons of L2/3 pyramidal neurons of Rasgrf2-2A-dCre;Camk2a-tTA;Ai78 mice. These Butterfly mice are a powerful tool for studying action potential and excitatory synaptic activity exclusively from the L2/3 pyramidal neuron network.
